# A causal glycerophospholipid–IL-18R1–CD9 axis connects lipid metabolism and T-cell activation in atopic dermatitis

**DOI:** 10.1093/bib/bbag187

**Published:** 2026-04-30

**Authors:** Ping-An Zhang, Jie-Lin Wang, Run-Dong Qin, Xiao-Nan Song, Ren-Ke Mo, Mei-Hua Dong, Xuan-Yu Pan, Jing Liu, Wan-Jun Wang, Shuo Chen, Jing Li

**Affiliations:** State Key Laboratory of Respiratory Disease, National Clinical Research Center for Respiratory Disease, Guangzhou Institute of Respiratory Health, Department of Allergy and Clinical Immunology, The First Affiliated Hospital of Guangzhou Medical University, 151 Yanjiangxi Rd, Guangzhou, Guangdong 510120, China; Department of Obstetrics and Gynecology, Department of Gynecologic Oncology Research Office, Guangzhou Key Laboratory of Targeted Therapy for Gynecologic Oncology, Guangdong Provincial Key Laboratory of Major Obstetric Diseases, Guangdong Provincial Clinical Research Center for Obstetrics and Gynecology, Guangdong-Hong Kong-Macao Greater Bay Area Higher Education Joint Laboratory of Maternal-Fetal Medicine, The Third Affiliated Hospital, Guangzhou Medical University, No. 63 Duobao Road, Liwan District, Guangzhou, Guangdong 510150, China; State Key Laboratory of Respiratory Disease, National Clinical Research Center for Respiratory Disease, Guangzhou Institute of Respiratory Health, Department of Allergy and Clinical Immunology, The First Affiliated Hospital of Guangzhou Medical University, 151 Yanjiangxi Rd, Guangzhou, Guangdong 510120, China; State Key Laboratory of Respiratory Disease, National Clinical Research Center for Respiratory Disease, Guangzhou Institute of Respiratory Health, Department of Allergy and Clinical Immunology, The First Affiliated Hospital of Guangzhou Medical University, 151 Yanjiangxi Rd, Guangzhou, Guangdong 510120, China; State Key Laboratory of Respiratory Disease, National Clinical Research Center for Respiratory Disease, Guangzhou Institute of Respiratory Health, Department of Allergy and Clinical Immunology, The First Affiliated Hospital of Guangzhou Medical University, 151 Yanjiangxi Rd, Guangzhou, Guangdong 510120, China; State Key Laboratory of Respiratory Disease, National Clinical Research Center for Respiratory Disease, Guangzhou Institute of Respiratory Health, Department of Allergy and Clinical Immunology, The First Affiliated Hospital of Guangzhou Medical University, 151 Yanjiangxi Rd, Guangzhou, Guangdong 510120, China; State Key Laboratory of Respiratory Disease, National Clinical Research Center for Respiratory Disease, Guangzhou Institute of Respiratory Health, Department of Allergy and Clinical Immunology, The First Affiliated Hospital of Guangzhou Medical University, 151 Yanjiangxi Rd, Guangzhou, Guangdong 510120, China; State Key Laboratory of Respiratory Disease, National Clinical Research Center for Respiratory Disease, Guangzhou Institute of Respiratory Health, Department of Allergy and Clinical Immunology, The First Affiliated Hospital of Guangzhou Medical University, 151 Yanjiangxi Rd, Guangzhou, Guangdong 510120, China; State Key Laboratory of Respiratory Disease, National Clinical Research Center for Respiratory Disease, Guangzhou Institute of Respiratory Health, Department of Allergy and Clinical Immunology, The First Affiliated Hospital of Guangzhou Medical University, 151 Yanjiangxi Rd, Guangzhou, Guangdong 510120, China; Department of Obstetrics and Gynecology, Department of Gynecologic Oncology Research Office, Guangzhou Key Laboratory of Targeted Therapy for Gynecologic Oncology, Guangdong Provincial Key Laboratory of Major Obstetric Diseases, Guangdong Provincial Clinical Research Center for Obstetrics and Gynecology, Guangdong-Hong Kong-Macao Greater Bay Area Higher Education Joint Laboratory of Maternal-Fetal Medicine, The Third Affiliated Hospital, Guangzhou Medical University, No. 63 Duobao Road, Liwan District, Guangzhou, Guangdong 510150, China; State Key Laboratory of Respiratory Disease, National Clinical Research Center for Respiratory Disease, Guangzhou Institute of Respiratory Health, Department of Allergy and Clinical Immunology, The First Affiliated Hospital of Guangzhou Medical University, 151 Yanjiangxi Rd, Guangzhou, Guangdong 510120, China

**Keywords:** atopic dermatitis, inflammatory proteins, metabolomics, CD9, IL-18R1, Mendelian randomization analysis

## Abstract

Atopic dermatitis (AD) involves complex metabolic–immune dysregulation, but the molecular links remain unclear. This study integrates a multilevel analytical framework to systematically investigate the metabolic–immune crosstalk in AD. Using linkage disequilibrium score regression and a two-step Mendelian randomization approach, we established genetic correlations and inferred causal relationships between plasma metabolites and inflammatory proteins, identifying 1-palmitoyl-2-arachidonoyl-GPC (PA-GPC) as a protective metabolite that exerts its effect primarily through downregulation of interleukin-18 receptor 1 (IL-18R1). Integration of single-cell transcriptomic data further revealed elevated IL-18R1 expression in T cells within the AD microenvironment and enabled stratification of T cells based on PA-GPC–associated metabolic activity, identifying 33 differentially expressed genes. Subsequent least absolute shrinkage and selection operator (LASSO) regression, combined with machine learning models and SHapley Additive exPlanations analysis, consistently prioritized CD9 as a key regulator. Functional validation showed that PA-GPC attenuates tumor necrosis factor-alpha (TNF-α)/interferon-gamma (IFN-γ)–induced inflammatory responses in human immortalized keratinocyte (HaCaT) cells and suppresses Th2 cytokine production in T cells. IL-18R1 knockdown reduced CD9 expression and Th2 cytokine production in T cells, whereas CD9 knockdown did not affect IL-18R1 expression, indicating that IL-18R1 acts upstream of CD9. Moreover, CD9 knockdown impaired T-cell viability, activation, and Th2 cytokine production. Collectively, these findings characterize metabolic–immune crosstalk in AD and identify a PA-GPC–IL-18R1–CD9 regulatory axis with potential therapeutic implications.

## Graphical Abstract

**Graphical Abstract:**
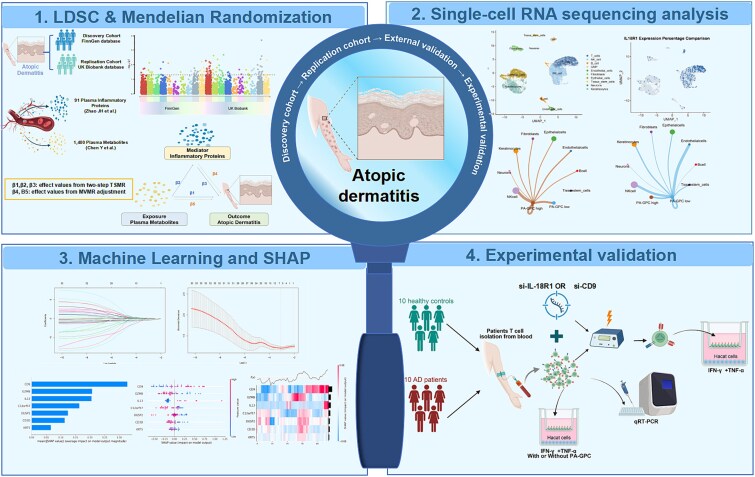

## Introduction

Atopic dermatitis (AD) is a chronic, relapsing, and pruritic inflammatory skin disorder that can affect individuals across all ages and ethnic groups, substantially reducing quality of life [1]. The disease is marked by persistent itching, xerosis, recurrent eczematous lesions, and disruption of the epidermal barrier [2–4]. Data from the Global Burden of Disease project reveal that AD impacts ~15%–20% of children and close to 10% of the adult population [5]. The disease significantly impacts patients’ quality of life by causing sleep disturbances, emotional distress, and social impairment [6].

The pathogenesis of AD has been attributed to multifaceted interactions involving genetic susceptibility, immune imbalance, and metabolic dysregulation [7]. With advances in untargeted and targeted metabolomics, diverse metabolites have been linked to the pathogenesis and severity of AD. For example, alterations in fatty acid composition can impair the skin barrier, promote microbial imbalance, and contribute to persistent inflammation [8]. In parallel, numerous immune-related cytokines and chemokines have been implicated in the pathophysiology of AD. Elevated levels of Th2 cytokines drive IgE production, eosinophil recruitment, and chronic inflammation [9, 10]. Additional cytokines such as IL-17, IL-22, and IL-18 also contribute to tissue damage and disease progression [11–13]. Notably, metabolism and immune function are not independent systems in AD, but are deeply interconnected [14]. Metabolic alterations can shape immune cell differentiation and cytokine production, while immune signals can feed back to modulate metabolic pathways. For example, IL-18 and IL-18R may affect lipid metabolism [15], while certain metabolic intermediates can promote or suppress immune activation [16]. This crosstalk forms a feedback loop that perpetuates inflammation and skin barrier dysfunction. Understanding the dynamic interaction between metabolic and immune networks is essential for identifying new biomarkers and therapeutic targets for AD. Given the complexity of AD, driven by the interaction of genetic, metabolic, and immunological elements, comprehensive and multidimensional research strategies are required to elucidate its underlying mechanisms.

Mendelian randomization (MR) has recently gained prominence as a tool for establishing causal associations by leveraging genetic variants as instruments, thereby minimizing confounding effects [17, 18]. It serves as a powerful framework for exploring the relationships between inflammatory markers, metabolites, and AD. At the same time, single-cell RNA sequencing (scRNA-seq) enables the discovery of distinct cellular contributors to disease, offering complementary insights into molecular and cellular pathways. Machine learning (ML) offers powerful capabilities for processing large-scale data and uncovering meaningful patterns, serving as an effective tool for decoding complex biological relationships. However, a major limitation of traditional ML models lies in their lack of interpretability, which poses a significant challenge in medical research. To overcome this limitation, SHapley Additive exPlanations (SHAP) provides an interpretive framework that measures how each variable contributes to predictive outcomes, enhancing both clarity and biological significance.

This study presents a multilevel integrative framework designed to systematically unravel the metabolic–immune crosstalk in AD. We first employed the linkage disequilibrium score regression (LDSC) and two-step MR approach to identify the genetic correlations and causal pathways between plasma metabolites and inflammatory proteins at the genetic level. To provide granular localization for these genetic signals, we then integrated single-cell transcriptomics to map relevant mediators to specific immune cell subsets within the AD skin microenvironment. Building upon this, ML algorithms combined with SHAP interpretation were applied to prioritize core hub genes within the identified cell populations. Finally, the biological significance of the resulting metabolic–immune axis was evaluated through a series of *in vitro* experiments and patient-derived cell assays. By integrating causal inference with high-resolution transcriptomics, this study offers a robust paradigm for identifying novel therapeutic targets in complex inflammatory diseases.

## Materials and methods

### Study design

This study implemented a causality-driven, multilevel integrative approach to systematically identify the metabolic–immune interactions underlying AD. To establish a robust genetic foundation, we first employed LDSC to assess the genetic correlations of inflammatory proteins and plasma metabolites with AD. Building upon these initial correlations, a two-step MR framework was applied to evaluate causal relationships and identify potential mediators. These analyses were conducted using the FinnGen discovery cohort and subsequently validated in the UK Biobank replication cohort, followed by multivariable Mendelian randomization (MVMR) to reduce confounding bias and confirm independent causal effects. All analyses were performed in line with the STROBE-MR standards [19].

To provide granular cellular resolution for these genetic findings, we integrated scRNA-seq data to characterize the immune microenvironment of AD. We observed that interleukin-18 receptor 1 (IL-18R1) expression was primarily localized to T cells, which prompted a focused downstream analysis of this population. To bridge systemic metabolic alterations with specific cellular functions, a metabolic score reflecting 1-palmitoyl-2-arachidonoyl-GPC (PA-GPC) biosynthesis was computed to stratify T cells into high or low activity groups. This stratification allowed us to identify differentially expressed genes (DEGs) and assess differences in cell–cell communication within a specific functional niche.

**Figure 1:**
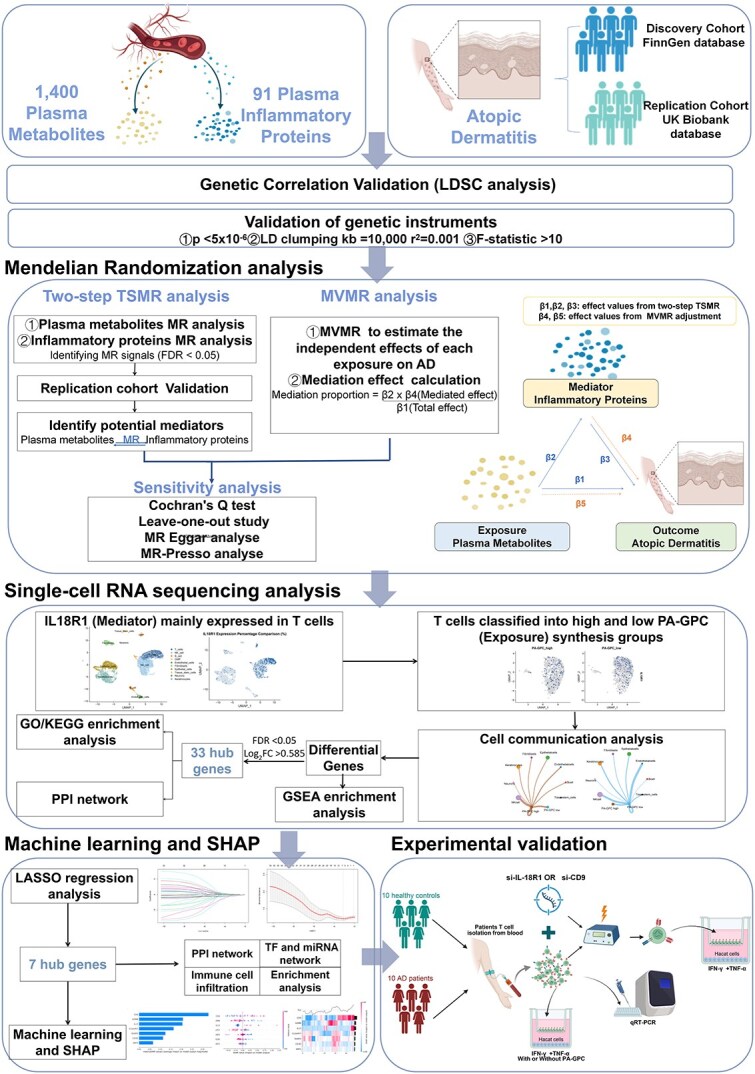
Overall study design of the article.

To further distill the most critical regulatory drivers from the resulting multiomics data, we utilized ML models including CatBoost, XGBoost, and NGBoost combined with SHAP interpretation. This process prioritized CD9 as the top-ranked predictor with the highest contribution to the model. The dynamic regulatory impact of CD9 was then explored using an in silico knockout approach. Finally, the biological significance of the identified PA-GPC/IL-18R1/CD9 axis was confirmed through functional assays. We utilized patient-derived T cells and a human immortalized keratinocyte (HaCaT) co-culture system to evaluate the anti-inflammatory effects, thereby validating the entire integrated discovery pipeline. An overview of the research framework is presented in Fig. 1.

### Data source

The discovery genome-wide association study (GWAS) dataset for AD was sourced from FinnGen [20]. Replication datasets were from the UK Biobank via the Open GWAS platform. We obtained GWAS summary statistics for 1400 plasma metabolites and 91 plasma inflammatory proteins from the GWAS Catalog [21, 22]. Because these datasets are derived from independent consortia, the likelihood of sample overlap is minimal.

In addition, the scRNA-seq dataset related to AD was retrieved from the Gene Expression Omnibus (GEO), with accession code GSE180885 [23]. For ML and SHAP analyses, GSE208405 [24] was used as the training dataset, and three cohorts (GSE256050 [25], GSE121212 [26], and GSE193309 [27]) served as external validation sets.

### Genetic correlation validation

Firstly, LDSC was applied as an exploratory approach to assess the genetic correlations of inflammatory proteins with AD and of plasma metabolites with AD. For each single nucleotide polymorphism (SNP), linkage disequilibrium (LD) scores were calculated to estimate association strength with complex traits under the principle of LD [28]. This step aimed to explore shared genetic features and broadly categorize potential signals prior to formal causal inference.

SNPs exhibiting a minor allele frequency <0.01 were filtered out. The calculated genetic correlations were presented along with their standard errors, and *P*-values under .05 were considered preliminary signals of genetic association [29]. Because this step was exploratory, no multiple-testing adjustment was undertaken, whereas subsequent MR analyses incorporated false discovery rate (FDR) correction. The “ldscr” package in R was applied for the analysis [30].

### Mendelian randomization analysis

The causal effect of 1400 plasma metabolites and 91 inflammatory proteins on AD was examined through a two-sample Mendelian randomization (TSMR) analysis. The MR framework is grounded on three essential premises: (i) the selected IVs must display a strong correlation with the exposure of interest; (ii) these IVs are required to be free from associations with potential confounders that might be related to the exposure–outcome relationship; and (iii) their effect on the outcome should operate only through the exposure pathway.

#### Selection of instrumental variables

SNPs linked to 1400 plasma metabolites and 91 inflammatory proteins were identified using a genome-wide cutoff of *P* < 5 × 10^−6^ [31, 32]. LD was assessed with the “clump data” method, applying a threshold of *r*^2^ < 0.001 and a window of 10 000 kb. To ensure independence, non-biallelic and correlated variants were excluded. Variants that remained were required to show a strong association with the exposure, and those with F-statistics below 10 were discarded. Subsequently, we screened all IVs against the GWAS Catalog to recognize and exclude any SNPs related to characterized confounders that could bias our results.

#### Mendelian randomization analysis workflow

A five-stage MR design was implemented in sequence to comprehensively examine causal associations between metabolites, inflammatory mediators, and AD. Crucially, this entire MR pipeline was conducted as a comprehensive and unbiased screen of all 1400 plasma metabolites and 91 inflammatory proteins, performed in parallel to, and independent of, our exploratory LDSC analysis. This approach ensures that no potential causal factors were prematurely excluded based on genetic correlation alone. The five stages were as follows:

Stage 1, Discovery TSMR Analysis. In the discovery phase, a TSMR was applied to evaluate associations of 1400 plasma metabolites and 91 inflammatory proteins with AD, using GWAS summary data from exposures and FinnGen outcomes. Significance was determined after FDR adjustment.

Stage 2, Replication TSMR Analysis. Associations that passed FDR correction in stage 1 were taken forward for replication using the UK Biobank AD GWAS dataset to confirm robustness.

Stage 3, Bidirectional MR for Mediation. To explore the causal direction and potential mediation pathways, we performed bidirectional TSMR between the replicated metabolite (PA-GPC) and inflammatory proteins (IL-18R1 and IL-18).

Stage 4, MVMR Analysis. The influence of correlated exposures was separated, and the independent causal role of each factor on AD was evaluated using MVMR with the FinnGen AD cohort as the outcome. We constructed three distinct models to assess the independent effects of (a) PA-GPC and IL-18R1, (b) PA-GPC and IL-18, and (c) PA-GPC, IL-18, and IL-18R1 combined.

Stage 5, Estimation of Mediation Effects. The mediation of PA-GPC on AD via IL-18R1 was evaluated using its effect on IL-18R1 (TSMR) and the direct effect of IL-18R1 on AD (MVMR model 2).

#### Comprehensive statistical methods in Mendelian randomization and mediation analysis

Potential causal associations between metabolites, inflammatory mediators, and AD were assessed through a two-step TSMR approach, followed by MVMR and mediation analyses. These MR analyses were conducted in R (v4.3.1) with the TwoSampleMR and MendelianRandomization packages [33]. We primarily determined causal associations using the inverse-variance weighted (IVW) method and complemented it with four alternative models, including MR-Egger, Weighted Median, Weighted Mode, and Simple Mode. We believe that the robustness of our results is significantly enhanced when the majority of these methods yield consistent results. When fewer than two SNPs were present, the Wald ratio was applied. Statistical significance was defined by FDR-adjusted *P*-values, and results were confirmed in a replication cohort. Only associations with consistent effects across discovery and replication sets and passing sensitivity checks were retained. In the second step, bidirectional MR was carried out to assess reciprocal associations between AD-related metabolites and inflammatory markers identified in step one.

In TSMR, β₁ denotes the overall impact of exposure on AD, β₂ the effect of exposure on the mediator, and β₃ the mediator’s effect on AD. When significance criteria were satisfied, MVMR was applied to disentangle the effects of each factor (e.g. PA-GPC, IL-18, IL-18R1) on AD, reducing confounding. IVW served as the main estimator, and in the multivariable setting, β₄ represented the mediator’s influence on AD. Mediation was quantified via the product of coefficients (β₂ × β₄). The direct effect was obtained by removing the mediation component from the total effect, with the mediated proportion expressed as: (mediation/total) × 100% [31, 34].

#### Sensitivity analysis

Horizontal pleiotropy was evaluated by MR-Egger and MR-Presso, where *P* > .05 indicated no evidence [35, 36]. Cochran’s Q test was applied to assess heterogeneity, with *P* > .05 interpreted as the absence of heterogeneity [37]. A leave-one-out procedure was further conducted by sequentially excluding individual SNPs to test their impact on the MR estimates [38].

#### Metabolic pathways enrichment analysis

Plasma metabolites identified from the MR analysis with IVW *P*-values <.05 were subjected to Kyoto Encyclopedia of Genes and Genomes (KEGG) enrichment analysis through MetaboAnalyst 6.0 (https://www.metaboanalyst.ca/) [39], where *P* < .05 was considered significant.

#### Bayesian co-localization analysis

Bayesian co-localization was assessed with the “coloc” R package [40] to test whether IL18R1, PA-GPC, and AD share a causal variant. Five scenarios were evaluated: no causal variant in the locus (H0), one affecting IL18R1 or PA-GPC (H1), one specific to AD (H2), independent variants for each trait (H3), or a common variant influencing both (H4). The coloc.abf algorithm was applied to calculate the posterior probability for H4 (PPH4). Values of PPH4 ≥ 0.8 indicated strong evidence of co-localization, whereas values between 0.5 and 0.8 were considered moderate. Visualization of regional co-localization was performed with the “LocusCompareR” package [41].

### Single-cell RNA sequencing

#### Single-cell RNA sequencing analysis processing

An scRNA-seq dataset associated with AD was acquired from the GEO database (accession: GSE180885) [23]. The raw data for each sample were processed to create a Seurat object using the Read10x function in the Seurat package (version 4.4.0) [42]. During preprocessing, cells with >10% mitochondrial transcripts or fewer than 500 or ˃4000 detected genes were excluded. Only high-quality cells meeting these thresholds were retained for integration. Normalization was carried out with the NormalizeData function (scale factor 10 000). Highly variable genes (*n* = 3000) were detected using the FindVariableFeatures function. These highly variable genes were then centered and scaled with the ScaleData function, followed by principal component analysis for dimensionality reduction. To eliminate batch effects, the Harmony algorithm was applied across datasets [43].

Uniform Manifold Approximation and Projection (UMAP) was applied to the first 15 Harmony-adjusted dimensions for visualization and dimensionality reduction. Cells were clustered through the k-nearest neighbor algorithm at a resolution of 1.0. This resolution parameter was empirically optimized to ensure sufficient granularity for identifying distinct immune cell subsets while maintaining cluster stability. The analysis yielded 19 clusters, which were then annotated into 10 major cell types using SingleR [44].

#### Investigation of 1-palmitoyl-2-arachidonoyl-GPC metabolic status and differential gene expression in T cells

Based on our MR findings, which suggested that PA-GPC may reduce AD risk by downregulating IL-18R1 expression, we examined the expression of IL-18R1 across all cell types. The highest expression was observed in T cells, leading to their selection for subsequent analysis. To assess PA-GPC-related metabolic activity in T cells, genes involved in PA-GPC biosynthesis were extracted from the glycerophospholipid metabolism pathway in the KEGG database. The AddModuleScore function was applied to compute PA-GPC metabolic scores for individual T cells. Based on the median score, T cells were stratified into PA-GPC_high and PA-GPC_low groups. DEGs were determined using FindMarkers (Wilcoxon test) with thresholds of FDR < 0.05 and |log_2_FC| > 0.585.

#### Cellular communication

The CellChat package (v1.6.1) was used to visualize interactions between PA-GPC_high/PA-GPC_low groups and other cell types based on scRNA-seq data [45]. The CellChatDB.human database was applied to predict intercellular interactions, using *P* < .05 as the threshold.

### Machine learning

#### Construction of training/validation sets

GSE208405 was chosen as the training set, including blood samples from 40 healthy controls and 87 AD patients (treatment-naïve or untreated). Using the IOBR package, probe IDs were converted to corresponding gene symbols [46]. GSE256050 served as the validation dataset, containing samples from 20 healthy donors and 28 AD patients [25]. Model robustness was further examined using two independent external cohorts, GSE121212 [26] and GSE193309 [27]. Common genes from training and validation sets were integrated into a single dataset for batch correction, after which the adjusted data were used for further analysis and modeling.

#### Machine learning and SHapley Additive exPlanations method

Using the glmnet package in R, least absolute shrinkage and selection operator (LASSO) regression was performed on the candidate genes to screen for key hub genes [47]. Afterward, three gradient boosting algorithm models: CatBoost [48], XGBoost [49], and NGBoost [50] were used for data training and prediction. These specific algorithms were selected due to their superior performance in handling high-dimensional biological data and their robustness against overfitting compared to traditional linear models. Receiver operating characteristic curves were plotted and area under the curve values calculated to assess model performance. Furthermore, to interpret the prediction results, we applied the SHAP method. SHAP values, based on cooperative game theory, assign an importance value to each feature to quantify its impact on the model output, thereby enhancing model interpretability [51]. Global feature importance was evaluated using a SHAP summary bar plot, in which importance was defined as the mean of absolute SHAP values [mean(|SHAP value|)] across all samples.

### Functional and network analyses

#### Enrichment analysis and immune infiltration analysis

Differentially expressed genes (*P* < .05) between PA-GPC_high and PA-GPC_low were first identified and then subjected to gene set enrichment analysis (GSEA) [52]. An adjusted *P*-value threshold of .05 defined statistical significance, and the 10 leading results were retained according to the directionality of the normalized enrichment scores. KEGG and Gene Ontology (GO) pathway enrichment analyses were performed on the 33 DEGs (FDR < 0.05 and |log_2_FC| > 0.5) and the 7 hub genes identified through LASSO regression. Immune infiltration of the hub genes was analyzed using the IOBR package.

#### Network analysis

The NetworkAnalyst platform (https://www.networkanalyst.ca/) was used to construct the protein-protein interaction (PPI) network, predict TF interactions, and analyze miRNA–gene interactions [53].

#### In silico knockout analysis

An in silico knockout analysis was conducted using scTenifoldKnk (v1.0.183) in R to investigate the biological significance of CD9 [54, 55]. Using the same AD scRNA-seq dataset as in previous analyses, a single-cell gene regulatory network (scGRN) was first constructed. CD9 expression was then computationally set to zero within this network to generate a corresponding “pseudo-knockout” scGRN. By comparing the pseudo-knockout scGRN with the original, we identified perturbed genes that were significantly affected by the virtual deletion of CD9. GO and KEGG enrichment analyses were performed on these genes to reveal biological processes and signaling pathways influenced by the in silico knockout of CD9.

## Results

### Genetic correlations between plasma metabolites, inflammatory factor, and atopic dermatitis

**Figure 2:**
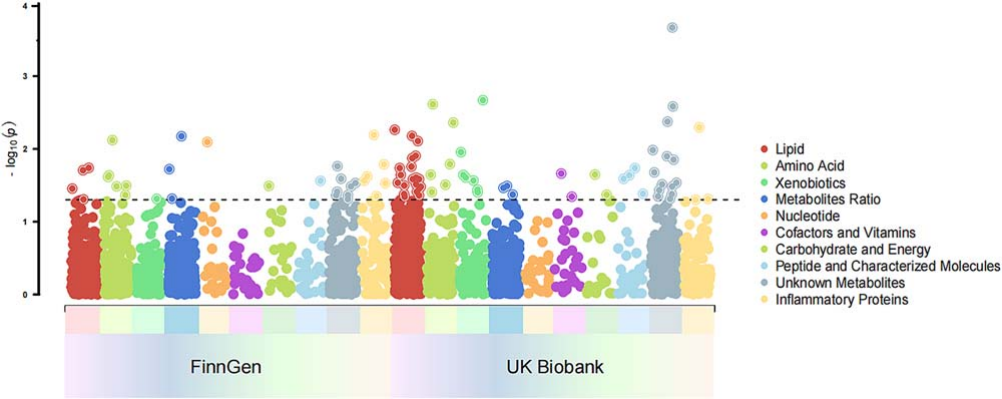
Genetic correlations between 91 circulating inflammatory proteins, 1400 plasma metabolites, and AD; the Manhattan plot shows the genetic correlations between 91 circulating inflammatory proteins, 1400 plasma metabolites, and AD.

We employed LDSC to explore the genetic correlations of plasma metabolites and inflammatory factors with AD in both the discovery and replication cohorts. In the discovery cohort, LDSC analysis identified 24 plasma metabolites significantly associated with AD, including 16 with negative and 8 with positive genetic correlations. In the replication cohort, 58 metabolites showed significant associations, with 41 negative and 15 positive correlations. In the discovery cohort, LDSC analysis revealed six inflammatory factors positively correlated with AD. In the replication cohort, three were significantly associated, including one negative and two positive correlations. To visually represent these findings, we have presented the results using a Manhattan plot (Fig. 2). Comprehensive results from the LDSC analysis can be found in Supplementary Tables S1 and S2.

### TSMR analysis of the causal effects of plasma metabolites and inflammatory factors on atopic dermatitis

**Figure 3:**
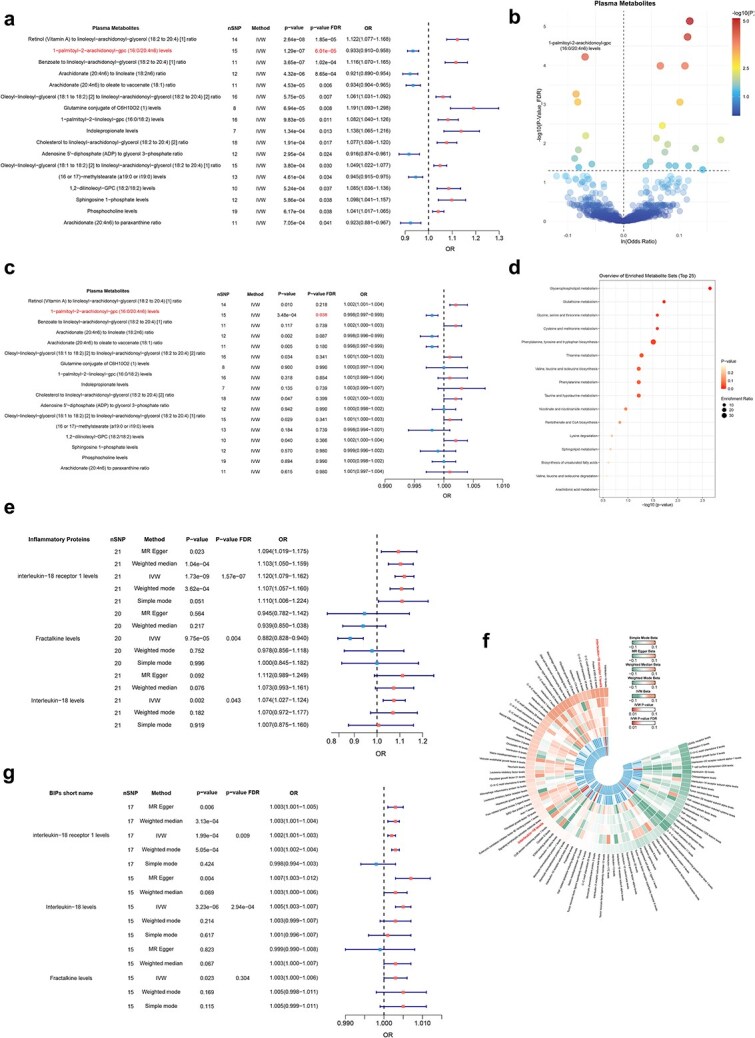
Causal associations of plasma metabolites and inflammatory proteins with AD across discovery and replication cohorts; (a) forest plot showing the causal effects of 1400 plasma metabolites on AD in the discovery cohort (FinnGen); these results identify putative metabolic drivers of AD risk; (b) volcano plot illustrating the distribution of effect sizes and significance levels for the metabolites in the discovery cohort; (c) forest plot showing associations between metabolites and AD in the replication cohort, confirming the robust protective effect of PA-GPC; (d) metabolite set enrichment analysis of metabolites with significant associations (P IVW <0.05) in the discovery cohort; (e) forest plot showing the causal effects of 91 circulating inflammatory proteins on AD in the discovery cohort; (f) circular heatmap representing inflammatory proteins associated with AD; (g) forest plot showing associations between inflammatory proteins and AD in the replication cohort, validating IL-18R1 as a significant risk factor.

A TSMR analysis was employed to evaluate whether circulating metabolites and inflammatory mediators exert causal influences on AD. In the discovery stage, 17 metabolites displayed statistically significant links with AD after FDR adjustment, of which 6 were related to a lower susceptibility and 11 to a higher susceptibility (Fig. 3a and b). Based on the enrichment analysis of metabolites with IVW *P*-values <.05, the most significantly enriched pathways include glycerophospholipid metabolism, glutathione metabolism, and amino acid-related pathways (Fig. 3d). These metabolites were subsequently evaluated in a replication cohort. Among them, PA-GPC (16:0/20:4n6) remained significantly associated with AD after FDR adjustment, with a consistent direction of effect (Fig. 3c). To ensure the robustness of this finding, we further compared five complementary MR models, which consistently supported the protective effect of PA-GPC (Supplementary Table S11). The detailed results of metabolites with a causal effect on AD are summarized in Supplementary Tables S3–S5.

In addition, three inflammatory factors demonstrated significant causal associations with AD after FDR correction in the discovery cohort, including IL-18R1 levels [odds ratio (OR), 95% confidence interval (CI): 1.120 (1.079–1.162), FDR-adjusted *P* = 1.57 × 10^−7^], fractalkine levels [OR (95% CI): 0.882 (0.828–0.940), FDR-adjusted *P* = .004], and IL-18 levels [OR (95% CI): 1.074 (1.027–1.124), FDR-adjusted *P* = .043] (Fig. 3e and f). Replication analyses confirmed the causal associations of IL-18R1 and IL-18 with AD after FDR correction (Fig. 3g). The stability of these causal links was further validated through the alignment of effect directions across multiple MR estimators (Supplementary Table S12). The detailed results of inflammatory factors with a causal effect on AD are summarized in Supplementary Tables S6–S8.

### Two-step two-sample and multivariable Mendelian randomization for mediation analysis

In the preceding analysis, TSMR and replication cohort validation revealed robust causal associations between PA-GPC, IL-18R1, IL-18, and AD. To explore the directionality of the causal relationships, a two-step TSMR analysis was performed between the metabolite PA-GPC and the inflammatory markers IL-18R1 and IL-18. The results showed that IL-18R1 and IL-18 had no significant causal effects on PA-GPC. In contrast, PA-GPC exhibited significant inverse causal effects on IL-18 [OR (95% CI): 0.960 (0.925–0.997), *P* = .032] and IL-18R1 [OR (95% CI): 0.9429 (0.9083–0.9789), *P* = .002], suggesting that PA-GPC may exert a protective role in AD pathogenesis by downregulating IL-18 and its receptor expression (Table 1).

**Table 1:** Summary of the results of the causal effect relationship between the metabolite PA-GPC and the inflammatory markers IL-18R1 and IL-18.

Exposure	Outcome	nSNP	*P*-value (IVW)	OR (95% CI)
IL-18	PA-GPC	22	.078	0.926 (0.850–1.0085)
IL-18R1	PA-GPC	22	.746	0.992 (0.942–1.044)
PA-GPC	IL-18	15	**.032**	0.960 (0.925–0.997)
PA-GPC	IL-18R1	15	**.002**	0.942 (0.908–0.979)

MVMR analysis was subsequently employed to explore the distinct contribution of each exposure to the outcome, with adjustments made for possible confounding influences. We constructed three distinct models to progressively test the mediation hypothesis (Table 2). In Model 1, which included PA-GPC and IL-18, PA-GPC retained a protective effect on AD (β = −0.058, *P* = .017) after adjusting for IL-18. This suggested that the protective mechanism of PA-GPC was largely independent of IL-18’s causal pathway. In Model 2, when PA-GPC and IL-18R1 were analyzed together, the effect of PA-GPC was attenuated to non-significance (*P* = .082), while IL-18R1 demonstrated a significant, direct risk effect on AD (β = 0.018, *P* = .001). This provided strong initial evidence that IL-18R1 acts as a mediator. Finally, to provide a more convincing result, we constructed a comprehensive Model 3 including all three factors. The results were conclusive: only IL-18R1 maintained a highly significant direct causal effect on AD (β = 0.023, *P* = 1.92 × 10^−4^), whereas the direct effects of both PA-GPC (*P* = .074) and IL-18 (*P* = .084) became non-significant. Collectively, this stepwise MVMR analysis provides robust evidence that the protective total effect of PA-GPC on AD is primarily mediated through its downregulation of IL-18R1. Based on the causal effect of PA-GPC on IL-18R1 (from TSMR) and the direct effect of IL-18R1 on AD (from MVMR model 2), we estimated the mediation effect of PA-GPC on AD pathogenesis via IL-18R1. Table 3 offers a concise summary of the findings.

**Table 2:** Results of multivariate Mendelian randomization analysis of the effects of PA-GPC and mediators (IL-18 or IL-18R1) on the onset of AD.

Model	Exposure	Outcome	nSNP	*P*-value (IVW)	β (IVW)	Standard error (IVW)
1	PA-GPC	AD	6	**.017**	−0.058	0.024
	IL-18	AD	6	.084	−0.095	0.055
2	PA-GPC	AD	2600	.082	−0.017	0.010
	IL-18R1	AD	2600	**.001**	0.018	0.005
3	PA-GPC	AD	2588	.074	−0.018	0.010
	IL-18	AD	2588	.084	−0.025	0.014
	IL-18R1	AD	2588	**.0002**	0.023	0.006

**Table 3:** The mediation proportion of IL-18R1 in the causal relationship between PA-GPC and AD.

Exposure	Mediator	Effect of exposure on outcome β_1_	TSMR effect of exposure on mediator β_2_	MVMR effect of mediator on outcome β_4_	Mediation effect (β_2_ × β_4_)	Direct effect	Mediated proportion (%) (β_2_ × β_4_)/β_1_
PA-GPC	IL-18R1	−0.069	−0.059	0.018	−0.001	−0.068	1.513%

**Figure 4:**
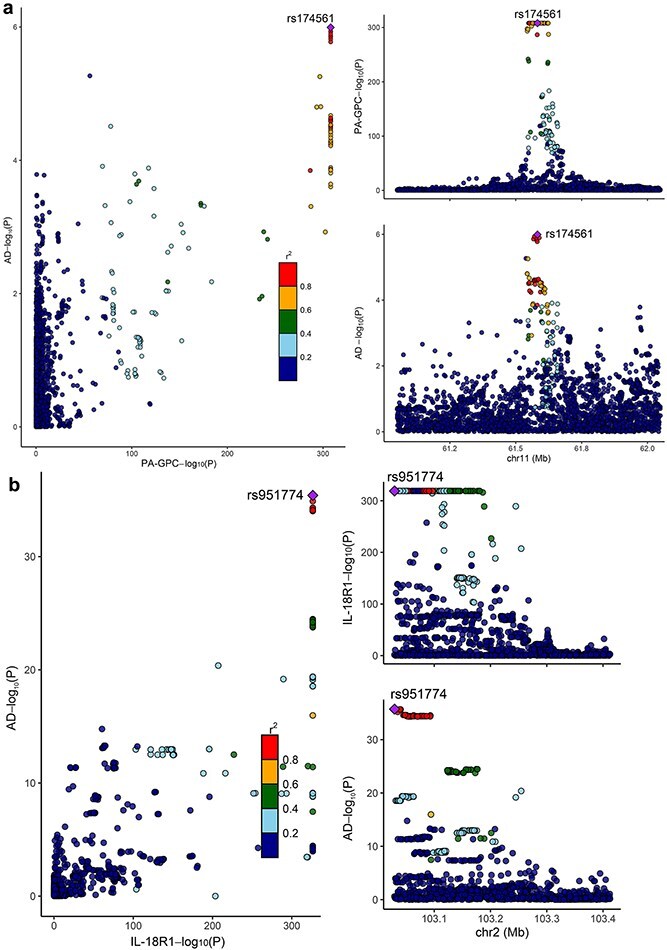
Co-localization analysis of molecular traits and atopic dermatitis (AD); (a) co-localization analysis of PA-GPC and AD; (b) co-localization analysis of IL-18R1 expression and AD.

Furthermore, co-localization analysis revealed that IL-18R1 had a high posterior probability of a shared causal variant with AD (PPH4 = 0.992) (Fig. 4a), while PA-GPC also showed supportive evidence (PPH4 = 0.792) (Fig. 4b). The results indicate that both traits could be involved in the development of AD through overlapping genetic signals.

### Single-cell transcriptomic analysis of 1-palmitoyl-2-arachidonoyl-GPC-associated cellular composition and intercellular communication

**Figure 5:**
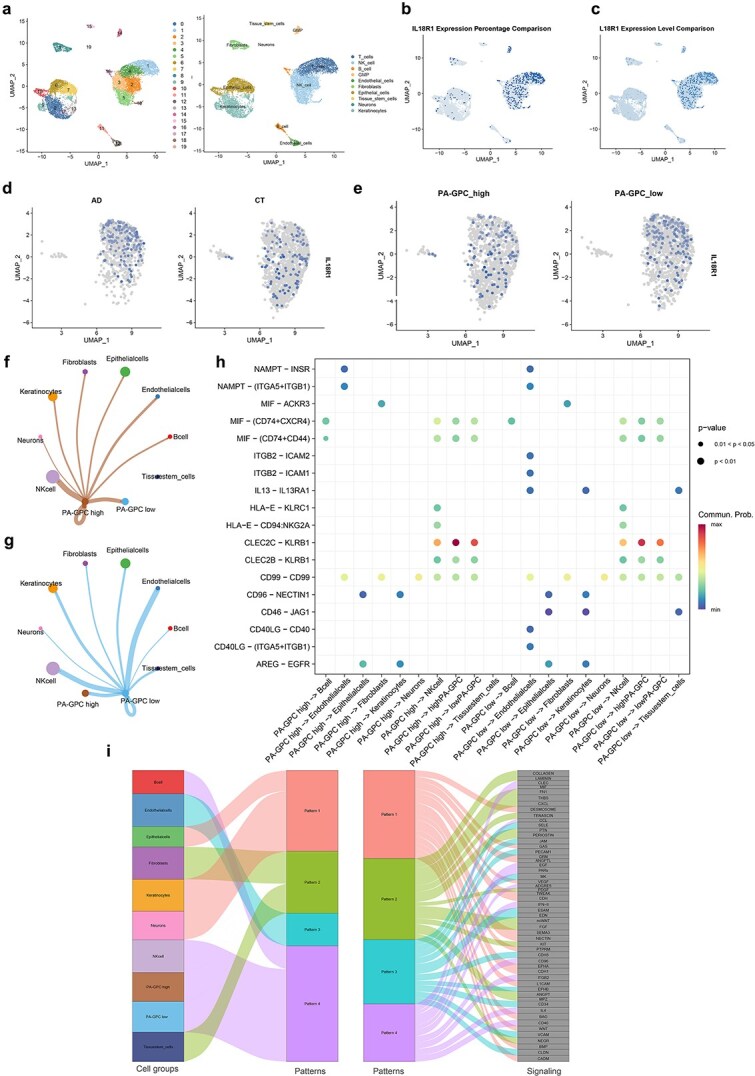
Single-cell RNA-seq analysis reveals IL-18R1 enrichment in T cells and its association with PA-GPC metabolic status; (a) UMAP plots showing cell clustering and annotation of 10 major cell types; (b–c) IL18R1 expression percentage and level across cell types, identifying T cells as the primary cellular source; (d) UMAP visualization of IL18R1 expression in T cells from AD patients versus healthy controls; the difference in expression between the two groups is statistically significant (Wilcoxon rank-sum test, *P* < .001); (e) IL18R1 expression in PA-GPC_high and PA-GPC_low T cells, showing that high PA-GPC activity correlates with reduced IL18R1 levels; (f–g) cell communication networks of PA-GPC_high (f) and PA-GPC_low (g) T cells with other cell types; (h) dot plot of key ligand–receptor interactions across different PA-GPC metabolic states; (i) Sankey diagram showing communication patterns and associated signaling pathways.

The potential role of PA-GPC in immune regulation within AD was assessed through scRNA-seq analysis of the GSE180885 dataset. Following rigorous quality filtering, 15 130 cells were preserved for subsequent analyses. A total of 19 cellular clusters were initially identified through unsupervised clustering (Fig. 5a, left). After annotation using canonical marker genes, these clusters were classified into 10 major cell types (Fig. 5a, right). Further analysis of the cellular distribution revealed that IL18R1 was expressed in both T cells and natural killer (NK) cells (Fig. 5b and c). However, T cells exhibited the most robust expression pattern and represented the predominant immune population in the AD microenvironment (Wilcoxon rank-sum test, *P* < .001). In T cells, IL18R1 expression was markedly elevated in AD patients relative to controls, as demonstrated by the Wilcoxon rank-sum test (*P* < .001) (Fig. 5d). We calculated a metabolic signature score utilizing genes associated with the PA-GPC biosynthetic pathway. Given that scRNA-seq does not directly measure metabolite levels, this gene signature-based scoring serves as a widely accepted proxy to infer the potential metabolic activity of PA-GPC within individual cells. Based on the median score, T cells were classified into PA-GPC_high and PA-GPC_low groups. This stratification allowed us to test whether the systemic causal link identified by MR (PA-GPC regulating IL-18R1) is reflected at the cellular level. Remarkably, IL18R1 expression exhibited a significantly higher level in the PA-GPC_low subset (Fig. 5e), suggesting that T cells with lower capacity for PA-GPC synthesis or processing are more prone to high IL-18R1 expression. This finding functionally validates the “PA-GPC–IL-18R1” axis specifically within the T-cell compartment.

Cell communication analysis revealed distinct signaling preferences between the two metabolic states. PA-GPC_high cells exhibited enhanced interactions with B cells and NK cells, particularly enriched in inflammatory chemotactic pathways such as MIF–CXCR4 and MIF–(CD74 + CD44) (Fig. 5f and h). In contrast, PA-GPC_low cells preferentially interacted with epithelial, stromal, and endothelial cells via adhesion- and barrier-related pathways, including CD40LG–(ITGA5 + ITGB1), CD40LG–CD40, CD46–JAG1, ITGB2–ICAM1/2, and IL13–IL13RA1 (Fig. 5g and h). These pathways are involved in immune cell recruitment and Th2-mediated inflammation, suggesting that PA-GPC_low cells may contribute to maintaining a pro-inflammatory microenvironment in AD. Further clustering of communication patterns identified four dominant signaling modules (Supplementary Fig. S1), among which Pattern 4 was closely associated with both PA-GPC_high/low cells and immune populations such as NK and B cells. This pattern was enriched in CD40, VCAM1, IL4, CD34, ITGB2, and CD96, which are involved in immune activation and cell adhesion (Fig. 5i).

### Differential genes and functional enrichment associated with 1-palmitoyl-2-arachidonoyl-GPC metabolic status

**Figure 6:**
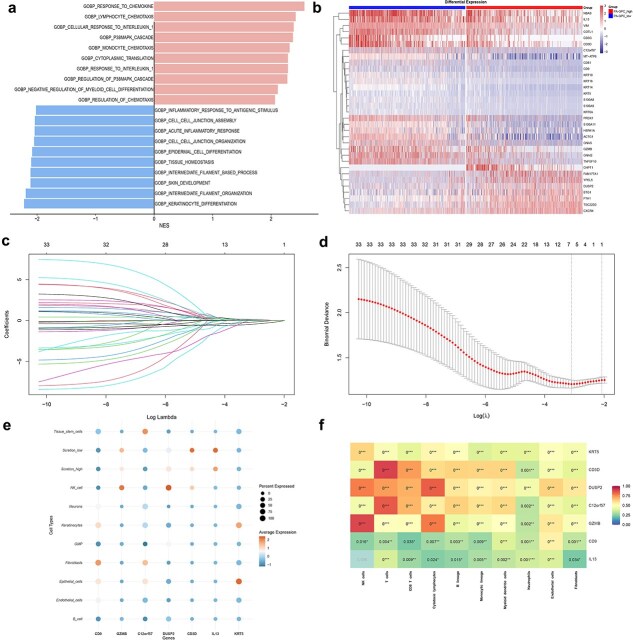
Identification and characterization of PA-GPC–related hub genes; (a) GSEA enrichment analysis of DEGs between PA-GPC_high and PA-GPC_low groups; (b) heatmap showing the expression of 33 DEGs (FDR < 0.05, |log_2_FC| > 0.585) between PA-GPC_high and PA-GPC_low groups; (c–d) LASSO regression analysis for hub gene selection from 33 DEGs; (e) expression patterns of the seven hub genes across major cell types; (f) correlation heatmap of hub genes with immune cell infiltration scores.

To further explore transcriptomic differences associated with PA-GPC metabolic status, we identified 2842 DEGs between the PA-GPC_high and PA-GPC_low groups. GSEA analysis revealed that these DEGs were positively enriched in immune and inflammatory pathways, including response to chemokines, lymphocyte, and monocyte chemotaxis, and response to interleukin-1, while they were negatively enriched in epithelial structure–related pathways, such as keratinocyte differentiation, skin development, cell–cell junction assembly, and tissue homeostasis (Fig. 6a). Applying standard filtering thresholds (FDR < 0.05 and |log_2_FC| > 0.5), we identified a core set of 33 DEGs, and their expression patterns are shown in the heatmap (Fig. 6b). PPI network analysis revealed functional interactions among these genes (Supplementary Fig. S2a). GO enrichment analysis (Supplementary Fig. S2b) showed that these genes were involved in intermediate filament organization, keratinocyte differentiation, and immune-related regulatory processes. Meanwhile, KEGG pathway analysis (Supplementary Fig. S2c) showed enrichment in immune and metabolic signaling pathways, including the IL-17 signaling pathway, Th1 and Th2 cell differentiation, estrogen signaling pathway, and gastric acid secretion. To visualize gene–function relationships, cnetplots were generated for both GO and KEGG results (Supplementary Fig. S2d and e), highlighting KRT14, IL13, and CD3D as key hub genes involved in multiple biological processes.

### LASSO-based identification of 1-palmitoyl-2-arachidonoyl-GPC-related hub genes

To further identify key regulatory genes, LASSO regression analysis was performed on the previously identified 33 differentially expressed genes, resulting in the selection of 7 hub genes (Fig. 6c and d). The distribution of these hub genes among distinct cell populations is presented in Fig. 6e. Immune infiltration analysis revealed that all identified genes were positively associated with immune cell infiltration, with particularly strong correlations observed for T cells, CD8+ T cells, and cytotoxic lymphocytes (Fig. 6f). GO enrichment analysis indicated that these genes were associated with a variety of immune-related biological processes (Supplementary Fig. S2f), such as complement-dependent cytotoxicity, positive thymic T-cell selection, and MAP kinase phosphatase activity. KEGG pathway analysis further confirmed that these hub genes were enriched in immune-related signaling pathways (Supplementary Fig. S2g), including T-cell receptor signaling pathways, Th1 and Th2 cell differentiation, and IL-17 signaling. The cnetplots (Supplementary Fig. S2h and i) illustrated the functional connections between these hub genes and their enriched GO and KEGG pathways. Additionally, PPI analysis (Supplementary Fig. S2j) showed interactions among these hub genes, while TF and miRNA co-regulatory network analysis (Supplementary Fig. S2k) revealed their potential regulatory relationships.

### Machine learning and SHapley Additive exPlanations reveal CD9 as a key predictor

To evaluate the predictive value and robustness of the seven identified hub genes, we constructed ML models using CatBoost, XGBoost, and NGBoost algorithms. The models were trained on the GSE208405 dataset and subsequently evaluated on three independent external validation cohorts to ensure the generalizability of our findings.

**Figure 7:**
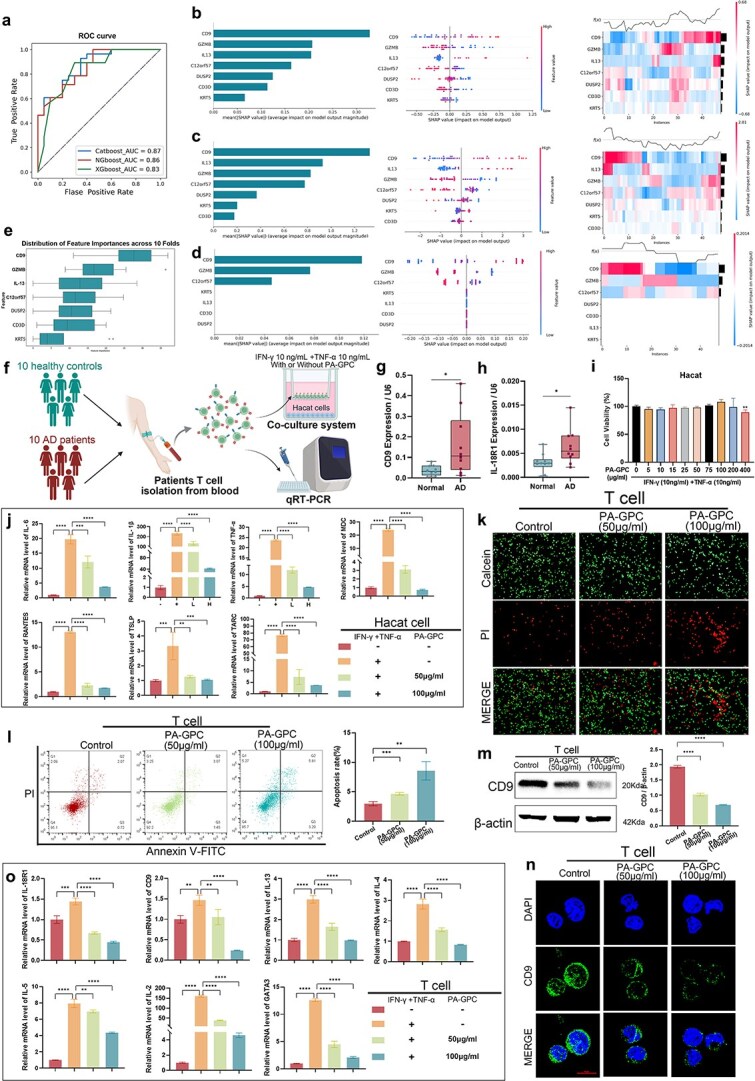
Machine learning identifies CD9 as a key hub gene and experimental validation confirms the anti-inflammatory effect of PA-GPC; (a) ROC curves for three machine learning models (CatBoost, NGBoost, XGBoost) based on hub gene expression in predicting AD; (b–d) SHAP analysis showing the importance of individual genes in the CatBoost (b), NGBoost (c), and XGBoost (d) models, including bar plots, beeswarm plots, and heatmaps; CD9 is consistently ranked as a top predictor; (e) distribution of feature importance across 10-fold cross-validation; (f) experimental workflow for validation using peripheral blood T cells from AD patients and healthy controls; (g–h) qPCR analysis of CD9 and IL-18R1 expression in T cells, showing upregulation in AD patients; (i) cytotoxicity assessment of PA-GPC in HaCaT cells; (j) expression of inflammatory mediators in HaCaT cells under tumor necrosis factor-alpha (TNF-α)/interferon-gamma (IFN-γ) stimulation with or without PA-GPC; (k) live/dead staining of T cells treated with PA-GPC; (l) annexin V/PI flow cytometry analysis of T cells; (m) western blot analysis of CD9 expression in T cells; (n) immunofluorescence staining of CD9 in T cells; (o) qPCR analysis of inflammatory and Th2-related cytokines and transcription factors in T cells; these results demonstrate that PA-GPC treatment effectively suppresses inflammatory responses and downregulates CD9.

In the first validation set, all models demonstrated robust performance, with AUC values of 0.87 for CatBoost, 0.86 for NGBoost, and 0.83 for XGBoost (Fig. 7a). To further interpret feature contributions, SHAP analysis was performed for each model, and summary, beeswarm, and heatmap plots were generated (Fig. 7b–d). In the second validation cohort, CatBoost, NGBoost, and XGBoost achieved AUC values of 0.81, 0.73, and 0.74, respectively (Supplementary Fig. S3a), with corresponding SHAP analyses shown in Supplementary Fig. S3b–d. In the third validation cohort, the AUC values were 0.77 for CatBoost, 0.75 for NGBoost, and 0.65 for XGBoost (Supplementary Fig. S4a), with the respective SHAP analyses presented in Supplementary Fig. S4b–d. As summarized in Supplementary Table S13, CD9 emerged as the top-ranked predictor in eight out of nine cohort-algorithm combinations across the three independent external datasets. Even when a minor rank fluctuation was observed in one specific model (NGBoost in the third cohort), the consistent prominence of CD9 across the majority of datasets and algorithms underscores its exceptional stability in AD. While the relative importance of lower-ranked genes varied slightly between different algorithms and cohorts, a central finding was exceptionally robust: CD9 consistently emerged as the top-ranked and most impactful predictor in virtually all analyses conducted. Other consistently high-ranking contributors included IL13, granzyme B (GZMB), and CD3D. To further assess model stability and the consistency of feature importance rankings, we performed 10-fold cross-validation within the training dataset, which confirmed that CD9 maintained its top-ranking status across all data folds (Fig. 7e). These results strongly support the critical role of these hub genes, particularly CD9, in the immune regulation and pathogenesis of AD.

### 1-palmitoyl-2-arachidonoyl-GPC attenuates tumor necrosis factor-alpha (TNF-α)/interferon-gamma (IFN-γ)–induced inflammatory responses in HaCaT cells

To experimentally validate the causal relationships suggested by our MR and transcriptomic analyses, we first isolated primary CD3^+^ T cells from the peripheral blood of 10 AD patients and 10 healthy controls for quantitative polymerase chain reaction (qPCR) analysis (primer sequences are provided in Supplementary Table S9). Supplementary Table S10 summarizes the clinical information and characteristics of the patients. In addition, a subset of AD-derived T cells was used in a co-culture system with HaCaT to assess anti-inflammatory activity (Fig. 7f). qPCR analysis revealed that T cells from AD patients expressed significantly higher levels of IL-18R1 and CD9 compared with healthy controls (Fig. 7g and h).

We next evaluated the cytotoxicity of PA-GPC on HaCaT. PA-GPC treatment exhibited minimal cytotoxic effects, with only a modest reduction in cell viability observed at the highest concentration (400 μg/ml) (Fig. 7i). Based on this result, 50 and 100 μg/ml were selected for subsequent experiments [56]. In the co-culture system, HaCaT cells were cultured together with T cells derived from AD patients that had been activated using a CD3/CD28 T cell activator, and upon stimulation with TNF-α and IFN-γ, the expression of inflammatory mediators—including MDC, RANTES, TSLP, TARCIL-6, IL-1β, and TNF-α—was markedly increased in HaCaT cells, whereas PA-GPC treatment significantly inhibited their induction in a dose-dependent manner, indicating that PA-GPC effectively attenuates TNF-α/IFN-γ induced inflammatory responses in keratinocytes (Fig. 7j). We subsequently evaluated the impact of PA-GPC on T-cell function. Live/dead staining and Annexin V/PI flow cytometry revealed that PA-GPC reduced T-cell viability and increased apoptosis in a concentration-dependent manner (Fig. 7k and l). Western blot and immunofluorescence analyses further demonstrated that CD9 protein expression in T cells was significantly downregulated following PA-GPC exposure, with greater suppression at higher concentrations (Fig. 7m and n). Finally, we examined the transcriptional changes of inflammatory factors in T cells within the co-culture system. TNF-α/IFN-γ stimulation strongly upregulated IL-18R1, CD9, IL-13, IL-5, IL-4, IL-2, and GATA3 expression, whereas PA-GPC treatment reversed these effects in a dose-dependent manner (Fig. 7o). Together, these findings provide functional validation that PA-GPC mitigates inflammatory responses.

### IL-18R1 functions upstream of CD9 to regulate T-cell activation in atopic dermatitis

**Figure 8:**
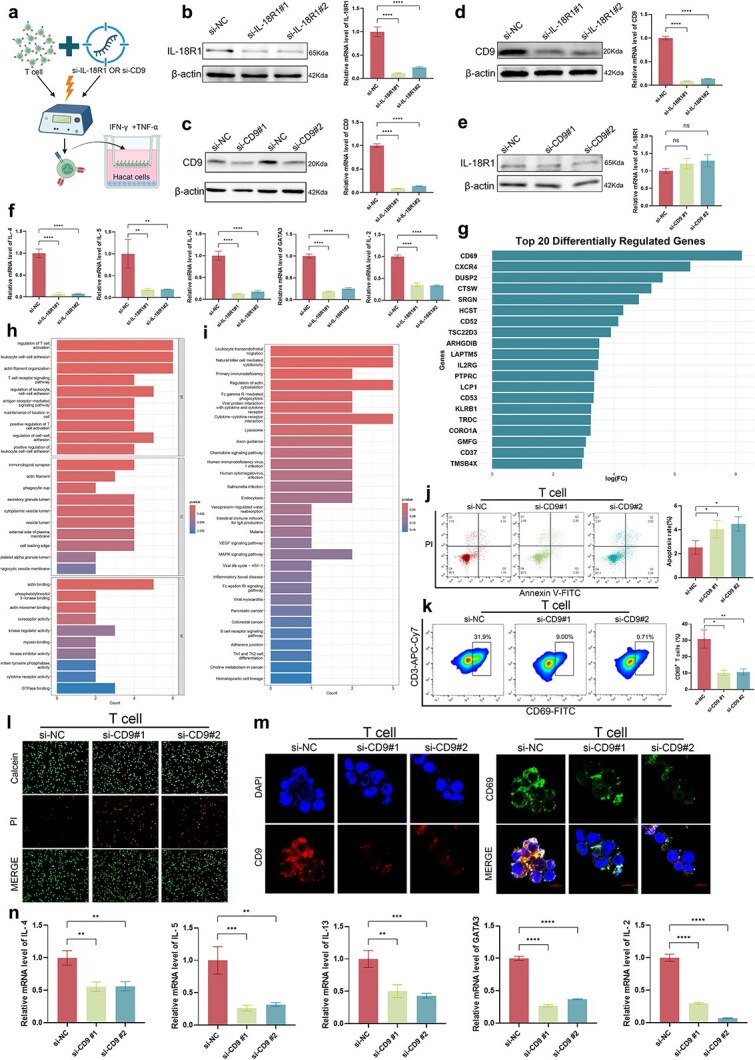
Functional implications of CD9 in T-cell activation and cytokine regulation; (a) experimental workflow of siRNA electroporation in primary T cells and subsequent analyses; (b–c) validation of IL-18R1 and CD9 knockdown efficiency by western blot and qPCR analyses; (d) assessment of CD9 expression at mRNA and protein levels following IL-18R1 knockdown; (e) assessment of IL-18R1 expression at mRNA and protein levels following CD9 knockdown; (f) qPCR analysis of IL-4, IL-5, IL-13, GATA3, and IL-2 expression in T cells following IL-18R1 knockdown; (g) top 20 differentially expressed genes identified following in silico knockout of CD9; (h) GO enrichment analysis of differentially expressed genes identified after in silico knockout of CD9; (i) KEGG pathway enrichment analysis following CD9 in silico knockout; (j) annexin V/PI flow cytometry assay of apoptosis in T cells after CD9 knockdown; (k) flow cytometry analysis of CD69+ T-cell proportions after CD9 knockdown; (l) live/dead staining of T cells after CD9 knockdown; (m) immunofluorescence staining of CD9 and CD69 in T cells; (n) qPCR analysis of IL-4, IL-5, IL-13, GATA3, and IL-2 expression in T cells following CD9 knockdown.

To experimentally validate the regulatory relationship between IL-18R1 and CD9, we performed siRNA-mediated knockdown experiments in primary human T cells. A schematic overview of the electroporation and co-culture workflow is shown in Fig. 8a. Efficient knockdown of CD9 and IL-18R1 was confirmed by qPCR and western blot analyses (Fig. 8b and c). Importantly, knockdown of IL-18R1 led to a significant downregulation of CD9 expression at both the mRNA and protein levels (Fig. 8d), followed by a marked reduction in Th2 cytokine production (Fig. 8f). In contrast, knockdown of CD9 did not affect IL-18R1 expression at either the mRNA or protein level (Fig. 8e), confirming the unidirectional regulatory relationship in which IL-18R1 functions upstream of CD9.

To investigate the dynamic regulatory impact of CD9, a silico knockout approach was applied. Virtual deletion of CD9 revealed several genes that were significantly dysregulated, including CD69, CXCR4, DUSP2, CTSW, and SRGN (Fig. 8g). GO enrichment analysis revealed a strong association with immune processes, particularly T-cell–related functions such as positive regulation of T-cell activation, T-cell activation, and T-cell receptor signaling (Fig. 8h). KEGG pathway enrichment further highlighted the involvement of CD9 in key immune regulatory pathways, including natural killer cell–mediated cytotoxicity, leukocyte transendothelial migration, and cytokine–cytokine receptor interactions (Fig. 8i).

Functionally, CD9 knockdown decreased T-cell vitality, as evidenced by Annexin V/PI staining (Fig. 8j) and live/dead cell assays (Fig. 8l). Flow cytometric analysis revealed a marked decrease in CD69^+^ T-cell proportions following CD9 knockdown (Fig. 8k). Consistently, immunofluorescence staining demonstrated reduced CD69 expression in CD9-deficient T cells (Fig. 8m). At the transcriptional level, qPCR analysis revealed that CD9 knockdown resulted in significant decreases in the expression of Th2-associated cytokines and transcription factors, including IL-4, IL-5, IL-13, IL-2, and GATA3 (Fig. 8n). Collectively, these findings biologically validate a directional IL-18R1–CD9 regulatory axis and demonstrate that CD9 acts as a functional downstream effector mediating T-cell activation and type 2 inflammatory responses in AD.

## Discussion

To our knowledge, this is the first investigation to integratively employ multiple bioinformatics approaches, including LDSC analysis, TSMR, MVMR, single-cell transcriptomics, ML, and SHAP, to systematically investigate the pathophysiology of AD. As a multiomics case study, the primary contribution of this work is the integration of these cross-scale analytical methods into a coherent and structured discovery pipeline. While the individual computational tools are well-established, their systematic combination offers a unique perspective on the lipid-immune interactions in AD. This stepwise strategy moves from population-level genetic causality to cell-specific localization and finally to molecular refinement. This structured approach reduces the risk of false-positive findings. It also provides a robust model for identifying potential biomarkers and therapeutic targets in complex inflammatory diseases.

Using LDSC analysis, we identified genetic correlations between multiple circulating metabolites, inflammatory proteins, and AD, suggesting that both metabolic and inflammatory factors are closely intertwined with the pathophysiological processes of AD. Further, TSMR analysis confirmed that 17 metabolites have a significant causal relationship with AD. Pathway enrichment analysis indicated that these metabolites are predominantly participating in glycerophospholipid metabolism. Glycerophospholipids (GPs) are essential structural components of cell membranes, playing critical roles in membrane fluidity, signal transduction, and immune activation [57]. Previous studies have shown that dysregulation of glycerophospholipid metabolism can influence the development of various immune-related diseases by modulating membrane receptor expression, activating signaling pathways, and altering the secretion of inflammatory cytokines [58–60]. In AD, previous metabolomic and lipidomic studies have identified distinct lipid profiles compared to healthy controls. Sakai *et al.* reported significant alterations in circulating phospholipids in AD patients [61]. Hotze *et al.* demonstrated that AD patients exhibiting a favorable response to omalizumab displayed significant changes in lipid metabolism, particularly an increase in glycerophospholipids, including phosphatidylcholine (PC) [62]. Similarly, Zhang *et al.* observed increased levels of PC following dupilumab treatment [63]. Lipid metabolism is likely to be critically involved not only in the pathogenesis of AD but also in modulating patients’ responsiveness to therapy. In this research, PA-GPC, as a PC subclass with immunomodulatory potential, showed a consistent suppressive association with AD across both discovery and replication cohorts.

Our two-sample MR analysis revealed that PA-GPC significantly reduced IL-18 and IL-18R1 levels, while MVMR analysis indicated that its inhibiting effect on AD is primarily mediated through IL-18R1 suppression. Crucially, the MVMR analysis revealed that IL-18 lost its significant association with AD after adjusting for IL-18R1, whereas IL-18R1 remained robust. This suggests that receptor availability serves as the rate-limiting bottleneck, confirming that these factors operate within a single vertical causal chain rather than through divergent pathways. A key finding from our mediation analysis was that the proportion of PA-GPC’s protective effect mediated by IL-18R1 was modest yet statistically significant, at 1.513%. This seemingly low percentage does not diminish the finding’s importance; rather, it underscores the profound biological complexity of AD. It is highly unlikely that the total effect of a single protective metabolite would be funneled through one molecular pathway. Instead, we interpret this 1.513% as a quantifiable “tip of the iceberg”—a specific, causally validated pathway that our robust analytical framework was able to successfully identify. The biological relevance of this specific axis is further substantiated by our single-cell transcriptomic findings. Guided by the MR results, our analysis of the AD cellular landscape revealed that in T cells, the primary expressors of IL18R1, there was a significant inverse relationship between their PA-GPC metabolic score and IL18R1 expression. This convergence of evidence from two independent analytical modalities, causal inference from genetic data and cellular expression patterns, provides powerful validation for the PA-GPC to IL-18R1 pathway as a genuine, albeit non-primary, contributor to AD pathogenesis.

To contextualize the therapeutic potential of PA-GPC, it is valuable to compare its proposed mechanism with that of established AD therapies. Milestone biologics like dupilumab function by directly blocking the shared receptor for IL-4 and IL-13, core cytokines driving type 2 inflammation in AD. Our study reveals a complementary pathway involving an upstream metabolic checkpoint. Our MR analysis identified a causal protective role for PA-GPC, mediated in part through the downregulation of IL-18R1. The IL-18/IL-18R1 axis has been proposed as an upstream regulator of TH2 cells, given its involvement in AD pathogenesis and its ability to promote T-cell activation [64–66]. Our preliminary *in vitro* experiments demonstrated that PA-GPC treatment of T cells co-cultured with epithelial cells significantly reduced the mRNA expression of IL-18R1, IL-4, and IL-13 in T cells. This preliminary evidence suggests that PA-GPC may exert dual immunomodulatory effects by both attenuating the IL-18R1 axis and suppressing key Th2 cytokines. These findings indicate that targeting PA-GPC may represent a novel strategy to intervene at the metabolic source of inflammation while simultaneously downregulating type 2 inflammation. Therefore, PA-GPC could potentially act synergistically when combined with downstream cytokine-blocking agents, such as dupilumab, offering new opportunities for combination therapy. However, these compelling hypotheses require further rigorous experimental validation. Future studies, including additional *in vitro* functional assays and *in vivo* experiments using AD animal models, will be essential to fully elucidate the molecular mechanisms of PA-GPC and confirm its therapeutic potential.

We next performed scRNA-seq analysis to further investigate the immunoregulatory role of PA-GPC and IL-18R1 in AD. IL18R1 expression was markedly upregulated in T cells. Subset analysis revealed that T cells with high PA-GPC metabolic activity exhibited significantly lower IL18R1 expression, consistent with our MR findings. Cell communication analysis showed that PA-GPC_low cells preferentially interacted with epithelial cells, keratinocytes, fibroblasts, and endothelial cells, engaging in adhesion- and tissue maintenance–related signaling pathways. Notably, ITGB2–ICAM1/2 are key pathways for leukocyte adhesion and transendothelial migration, indicating enhanced immune cell recruitment in inflamed tissues [67], while IL13–IL13RA1 is a hallmark Th2 inflammatory axis associated with barrier dysfunction and chronic inflammation [68]. The evidence points to PA-GPC_low cells may contribute to sustaining a pro-inflammatory microenvironment through enhanced cell adhesion and Th2 signaling.

Using LASSO regression, we identified seven key hub genes associated with PA-GPC metabolic status, all of which showed prominent expression in immune cells in AD patients. GO and KEGG enrichment analyses indicated that these genes are primarily participating in classical immune and inflammatory pathways, including Th1 and Th2 cell differentiation, T-cell receptor signaling, and the IL-17 signaling pathway. Previous studies have shown that AD pathogenesis involves Th2-skewed responses as well as contributions from Th1 and Th17 cells [26, 69]. The expression patterns of PA-GPC–associated hub genes closely align with these immune cell subsets, further supporting their central role in the metabolic–immune crosstalk underlying AD pathogenesis.

Across three validation cohorts, SHAP analyses of the three ML models consistently identified CD9, IL13, and GZMB as top contributors, indicating their potential involvement in the initiation and advancement of AD. IL13 is recognized as a key cytokine implicated in AD pathogenesis, known to regulate skin barrier function, promote IgE production, and recruit inflammatory cells [68]. GZMB, a cytotoxic effector released by T cells and NK cells, also is critically involved in inflammation-associated tissue damage in AD [70].

Critically, our study moves beyond descriptive associations by experimentally establishing the hierarchy of the identified metabolic–immune interface. While SHAP analysis prioritized CD9, our subsequent IL-18R1 knockdown experiments provided the biological validation. We demonstrated that CD9 is functionally downstream of IL-18R1, transforming the computational prediction into a verified biological mechanism.

CD9, belonging to the tetraspanin protein family, is critically involved in processes such as immune cell attachment, migration, immune synapse assembly, and exosome secretion [71, 72]. In T cells, CD9 is enriched at the immunological synapse (IS), which represents the interface where T cells engage with antigen-presenting cells during activation [72]. Within this region, CD9 interacts with other tetraspanins and integrins to regulate signaling events. For instance, it cooperates with the integrin LFA-1 on T cells, influencing its clustering and adhesive capacity at the IS [73]. Through these interactions, CD9 affects downstream signaling events essential for full T-cell activation. Indeed, CD9, along with tetraspanin CD151, accumulates at the T-cell side of the IS, and silencing CD9 significantly impairs T-cell activation signals. CD9 knockdown leads to reduced IL-2 cytokine secretion and lower expression of the early activation marker CD69 [74]. In our study, the virtual deletion of CD9 significantly perturbed pathways central to T-cell biology, including “T cell activation,” “T cell receptor signaling,” and “leukocyte cell–cell adhesion.” This was further supported by the significant dysregulation of key immune effector genes like the activation marker CD69 and the chemokine receptor CXCR4. Moreover, siRNA-mediated knockdown of CD9 in primary human T cells resulted in increased T-cell apoptosis and decreased CD69 expression, reinforcing its crucial role in T-cell activation.

CD9, when co-stimulated with CD28, enhances the expansion of naive T cells. This activation drives both CD4^+^ and CD8^+^ naive T cells toward type 2 effector phenotypes, leading to the secretion of Th2 cytokines [75]. In our experiments, CD9 knockdown in T cells suppressed Th2 cytokine secretion (IL-4, IL-5, IL-13), emphasizing its role in type 2 immunity. Earlier investigations indicated that CD9 contributes to endothelial–leukocyte interactions by co-distributing with adhesion molecules (e.g., intercellular adhesion molecule 1 [ICAM-1] and vascular cell adhesion molecule 1 [VCAM-1]) at the endothelial surface, thereby promoting leukocyte attachment and transendothelial passage [76]. CD9 knockdown was further observed to reduce ICAM-1 and VCAM-1 surface abundance, impairing leukocyte recruitment under inflammatory conditions [77]. These findings align with our virtual knockout results, which enriched pathways related to “leukocyte cell–cell adhesion.” Together, these results suggest that CD9 not only regulates leukocyte adhesion but may also play a role in Th2 immune polarization, a hallmark of AD. Given its pivotal involvement in immune regulation and inflammatory responses, CD9 is regarded as a potential therapeutic candidate for AD.

Our study is the first to integrate MR, multiomics analysis, single-cell transcriptomics, ML, and SHAP to systematically investigate the metabolites, inflammatory factors, and key pathways in AD. The MR design minimizes confounding bias and strengthens causal inference. Moreover, the integration of multiomics, ML, and SHAP approaches enabled the robust identification of hub genes associated with AD. However, several limitations should be acknowledged. One limitation of our research is that the GWAS summary statistics used were predominantly derived from populations of European descent. It is well-established that genetic architecture, allele frequencies, and LD patterns can differ substantially across global populations. Consequently, the IVs identified for PA-GPC and IL-18R1 may not have the same validity or effect size in non-European populations, and our findings may therefore not be directly generalizable. While we conducted a thorough search for suitable datasets to replicate our analysis in other ancestries, a critical barrier is the current lack of large-scale, publicly available GWAS for our specific exposures (plasma metabolites and inflammatory proteins) in ancestrally diverse cohorts, such as East Asian or African populations. Future studies in these populations are crucial to validate the causal axis identified in our work and to understand the full spectrum of metabolic–immune interactions in AD globally. In addition to these limitations related to population diversity, our study also has experimental limitations. Despite the promising results obtained from *in vitro* cell line models and computational analyses, the lack of comprehensive *in vivo* validation remains a significant gap. While we performed functional assays in cell lines, such experiments cannot fully replicate the complexity of the immune response and tissue microenvironment *in vivo*. Furthermore, the use of cell lines, while valuable for mechanistic insight, may not perfectly reflect the diversity of responses seen in primary patient cells or tissues. Further *in vivo* and human tissue investigations are needed to substantiate the therapeutic value of PA-GPC and to thoroughly examine its immune modulation in AD.

This study presents a comprehensive, multilayered analysis of AD by integrating MR, multiomics profiling, single-cell transcriptomics, and ML. We identified PA-GPC as a key protective metabolite and IL-18R1 as a critical inflammatory mediator. Importantly, CD9 was highlighted as a potential immunoregulatory factor in AD, functioning as a key connector between metabolic alterations and immune activation. CD9 emerges as a promising therapeutic candidate that warrants additional exploration in mechanistic and translational research.

Key Points

- 1-Palmitoyl-2-arachidonoyl-GPC (PA-GPC) was identified as a protective metabolite that may attenuate atopic dermatitis (AD) through IL-18R1 downregulation.
- Single-cell transcriptomics combined with machine learning and SHapley Additive exPlanations analyses identified CD9 as a key hub gene in AD T cells.
- *In vitro* experiments demonstrated that PA-GPC attenuated inflammatory responses in HaCaT cells and reduced Th2 cytokine production in T cells.
- IL-18R1 acted upstream of CD9, and IL-18R1 knockdown reduced Th2 cytokine production, while CD9 knockdown impaired T-cell viability, activation, and Th2 cytokine production.

## Supplementary Material

bbag187_Supplemental_Files

## Data Availability

The GWAS summary statistics used in this study are publicly available and were obtained from the FinnGen database (https://www.finngen.fi/en), the UK Biobank via the Open GWAS database (https://gwas.mrcieu.ac.uk), and the GWAS Catalog (accession numbers: GCST90274758 to GCST90274848 for inflammatory proteins, and GCST90199621 to GCST90201020 for plasma metabolites). The single-cell RNA-seq dataset (GSE180885) and the bulk RNA-seq datasets used for machine learning (GSE208405, GSE256050, GSE121212, GSE193309) are all available from the GEO database. All key analysis scripts associated with this article are publicly available in our GitHub repository: https://github.com/PAZworkspace/CD9_AD.
