# Supplementary material for: A causal glycerophospholipid–IL-18R1–CD9 axis connects lipid metabolism and T-cell activation in atopic dermatitis
Source: Brief Bioinform. 2026 Apr 30;27(2):bbag187. doi: 10.1093/bib/bbag187 (PMC13132603; doi:10.1093/bib/bbag187)
Supplement: bbag187_Supplemental_Files [file bbag187_supplemental_files.zip › Supporting_Information_bbag187.docx]

**A Causal Glycerophospholipid–IL-18R1–CD9 Axis Connects Lipid Metabolism and T-Cell Activation in Atopic Dermatitis**

Ping-An Zhang^#1^, MD, Jie-Lin Wang^#2^, PhD, Run-Dong Qin^1^, PhD, Xiao-Nan Song, PhD, Ren-Ke Mo, PhD, Mei-Hua Dong, PhD, Xuan-Yu Pan, MD, Jing Liu, MD, Wan-Jun Wang*^1^, PhD, Shuo Chen*^2^, PhD, Jing Li*^1^, MD

1. State Key Laboratory of Respiratory Disease, National Clinical Research Center for Respiratory Disease, Guangzhou Institute of Respiratory Health; Department of Allergy and Clinical Immunology, The First Affiliated Hospital of Guangzhou Medical University. 151, Yanjiangxi Rd, Guangzhou, Guangdong, 510120, China

2. Department of Obstetrics and Gynecology, Department of Gynecologic Oncology Research Office; Guangzhou Key Laboratory of Targeted Therapy for Gynecologic Oncology; Guangdong Provincial Key Laboratory of Major Obstetric Diseases; Guangdong Provincial Clinical Research Center for Obstetrics and Gynecology; Guangdong-Hong Kong-Macao Greater Bay Area Higher Education Joint Laboratory of Maternal-Fetal Medicine; The Third Affiliated Hospital, Guangzhou Medical University, Guangzhou, China.

# These authors contributed equally to this work

Ping-An Zhang, Email: zhang_pa@126.com

Jie-Lin Wang, Email: kilam1@foxmail.com

*Co-corresponding author:

Jing Li, Email: jingli1016@vip.163.com; Address: No.151, Yanjiangxi Road, Yuexiu District, Guangzhou, Guangdong, 510120, China

Shuo Chen, Email: chenshuo077003@163.com; Address: No.63 Duobao Road, Liwan District, Guangzhou City, Guangdong Province, P.R. China

Wan-Jun Wang, Email: wanwang506@163.com; Address: No.151, Yanjiangxi Road, Yuexiu District, Guangzhou, Guangdong, 510120, China

**Experimental Methods**

**Reagents**

PA-GPC was obtained from Shanghai Yuanye Bio-Technology Co. (Shanghai, China). Cytokines IFN-γ, TNF-α, and IL-2 were obtained from Yeasen Biotechnology (Shanghai, China). Human CD3⁺ T Cell Isolation Kit (Negative Isolation) was purchased from Yeasen Biotechnology. Primers were synthesized by BGI Genomics Co. (Shenzhen, China). Antibodies against CD9 (60232-1-Ig; Proteintech) and CD69 (AF6435; Beyotime) were used. Anti-CD3 and anti-CD69 antibodies were supplied by BD Pharmingen (USA). ImmunoCult™ CD3/CD28 T Cell Activator was supplied by STEMCELL Technologies.

**Cell culture and transfection**

HaCaT cells were obtained from Guangzhou Lige Technology Co., Ltd. (Guangzhou, China) and maintained in high-glucose DMEM (Thermo Fisher Scientific) containing 10% FBS (ExCell Bio) and 1% penicillin–streptomycin.

Peripheral venous blood samples were collected from 10 patients with AD and 10 healthy controls (without allergic disease). PBMCs were isolated using Lymphocyte Separation Medium (Yeasen, 40503ES60). Using the Human CD3⁺ T Cell Isolation Kit (Yeasen), CD3⁺ T cells were purified, and RNA was subsequently isolated for downstream qPCR. For stimulation and proliferation, T cells from an AD patient were cultured in RPMI-1640 medium (Thermo Fisher Scientific) with 10% FBS, 1% penicillin–streptomycin, IL-2 (10 μg/mL), and CD3/CD28 T Cell Activator (STEMCELL).

For siRNA transfection, T cells (2 × 10⁶) were electroporated with siRNA (1 µM) using a BTX ECM830 electroporation system (200 V, 30 ms). si-CD9 (#1 and #2), si-IL-18R1 (#1 and #2) and control siRNAs were purchased from BioSune Biotechnology (Shanghai, China). qPCR and western blotting confirmed knockdown efficiency at 48 h post-transfection.

**Co-culture and anti-inflammatory activity assay**

HaCaT cells (7 × 10⁵/mL) were seeded into 6-well plates and cultured for 24 h until reaching 80–90% confluence. CD3/CD28 activated si-CD9–transfected (or si-IL-18R1 or si-NC control) T cells (5 × 10⁵ per insert) were then placed in the upper chambers of 0.4-µm Transwell inserts, with HaCaT cells maintained in the lower chambers. Both compartments were treated with IFN-γ and TNF-α (10 ng/mL each), with or without PA-GPC (0, 50, or 100 μg/mL). After 12 h of co-culture, HaCaT cells and T cells were collected for downstream analyses.

**Cell proliferation**

HaCaT cells (5,000 per well) were plated in 96-well plates, treated with or without PA-GPC for 24 h, and subsequently incubated with 10 μL CCK-8 reagent (Yeasen) for 2 h. The absorbance was then recorded at 450 nm.

**Apoptosis assay**

T cells were collected, rinsed with PBS, and resuspended in 100 μL Annexin V binding buffer containing 5 μL Annexin V-FITC and 5 μL PI (BD Pharmingen). After 20 min of dark incubation, and apoptosis was assessed by flow cytometry.

**Quantitative real-time PCR**

Using TRIzol reagent (1 mL; Takara), total RNA was extracted, washed with 75% ethanol after chloroform and isopropanol precipitation, and finally dissolved in DEPC-treated water. RNA concentration was measured by spectrophotometry. A reverse transcription kit (Yeasen) was used for cDNA synthesis, followed by qPCR with Yeasen SYBR qPCR Master Mix. The sequences of primers are presented in Supplementary Table 9.

**Calcein/PI cell viability assay**

T cells were incubated in a Calcein AM/PI solution (Beyotime) at 37 °C for 30 min, and mounted on slides. Fluorescence was observed under a microscope.

**Immunofluorescence staining**

T cells were fixed with 4% paraformaldehyde for 30 min, mounted on poly-L-lysine–coated coverslips, and dried. 5% BSA was used to block the cells for 1.5 h. Cells were incubated overnight at 4 °C with primary antibodies (anti-CD9 or anti-CD69), followed by exposure to Alexa Fluor 488- or 594-labeled secondary antibodies (Proteintech) for 1 h in the dark. Images were collected using confocal microscopy.

**Western blot**

Proteins were isolated using RIPA buffer and quantified through a BCA assay. Equal amounts (30 µg) were resolved by SDS-PAGE and transferred onto PVDF membranes. After 5% BSA blocking for 2 h, the membranes were incubated with primary antibodies (anti-CD9, anti-IL-18R1 or anti-β-Actin), followed by HRP-conjugated secondary antibodies. Images were collected using the Bio-Rad imaging system.

**Statistical analysis**

All statistical analyses were performed in GraphPad Prism 9.5. Results are shown as mean ± SEM. Group differences were analyzed using one-way ANOVA followed by Dunnett’s post hoc test for multiple comparisons with the control group. For qPCR of patient-derived T cells, the Mann–Whitney test was applied.

**Supplementary Figures**


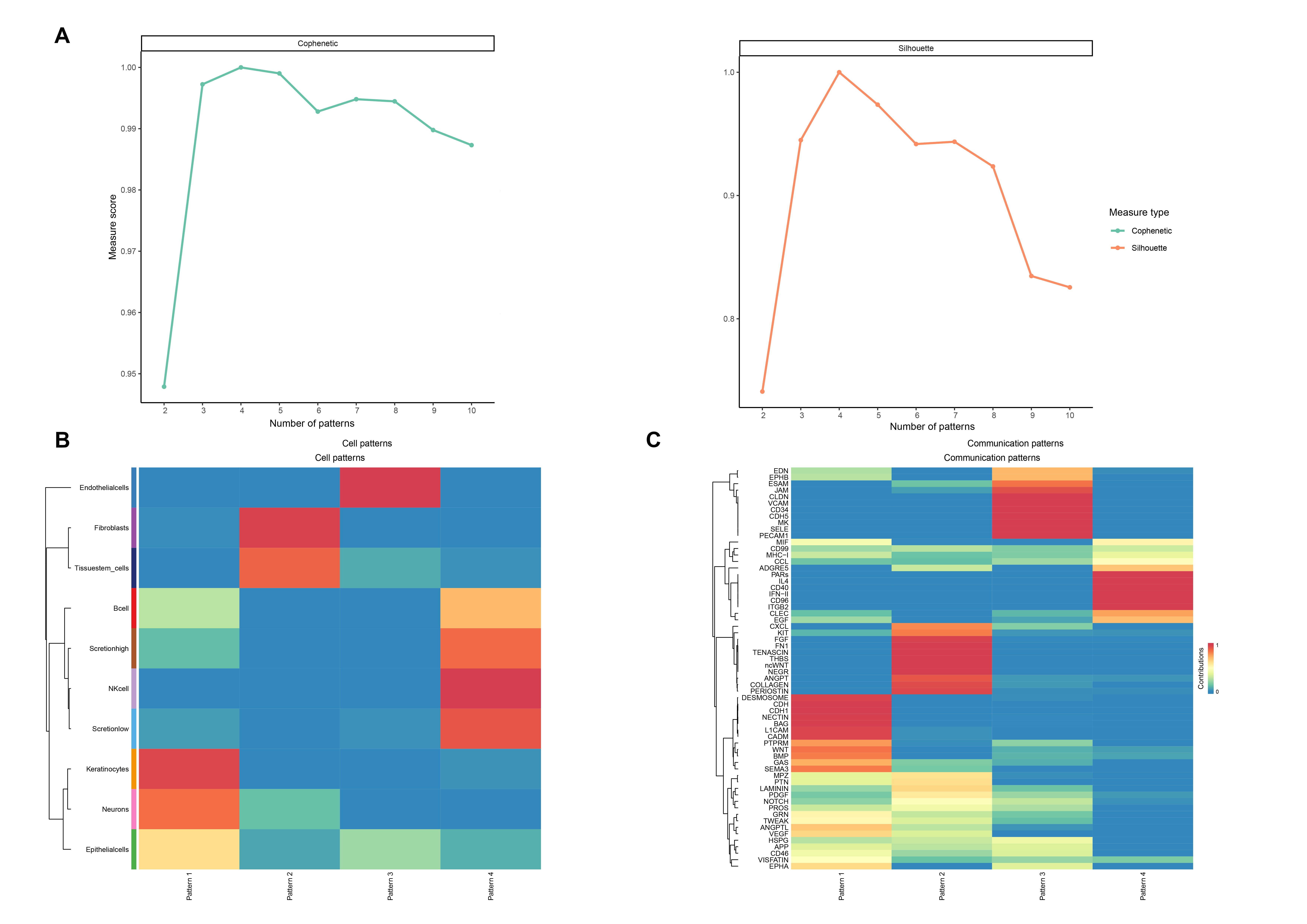


**Figure S1. Identification of four dominant signaling modules in cell–cell communication patterns.**

**(A)** Cophenetic and silhouette scores for different numbers of communication patterns used to determine the optimal clustering.

**(B)** Heatmap showing the distribution of cell type contributions across the four identified communication patterns.

**(C)** Heatmap of signaling pathway contributions within each communication pattern.


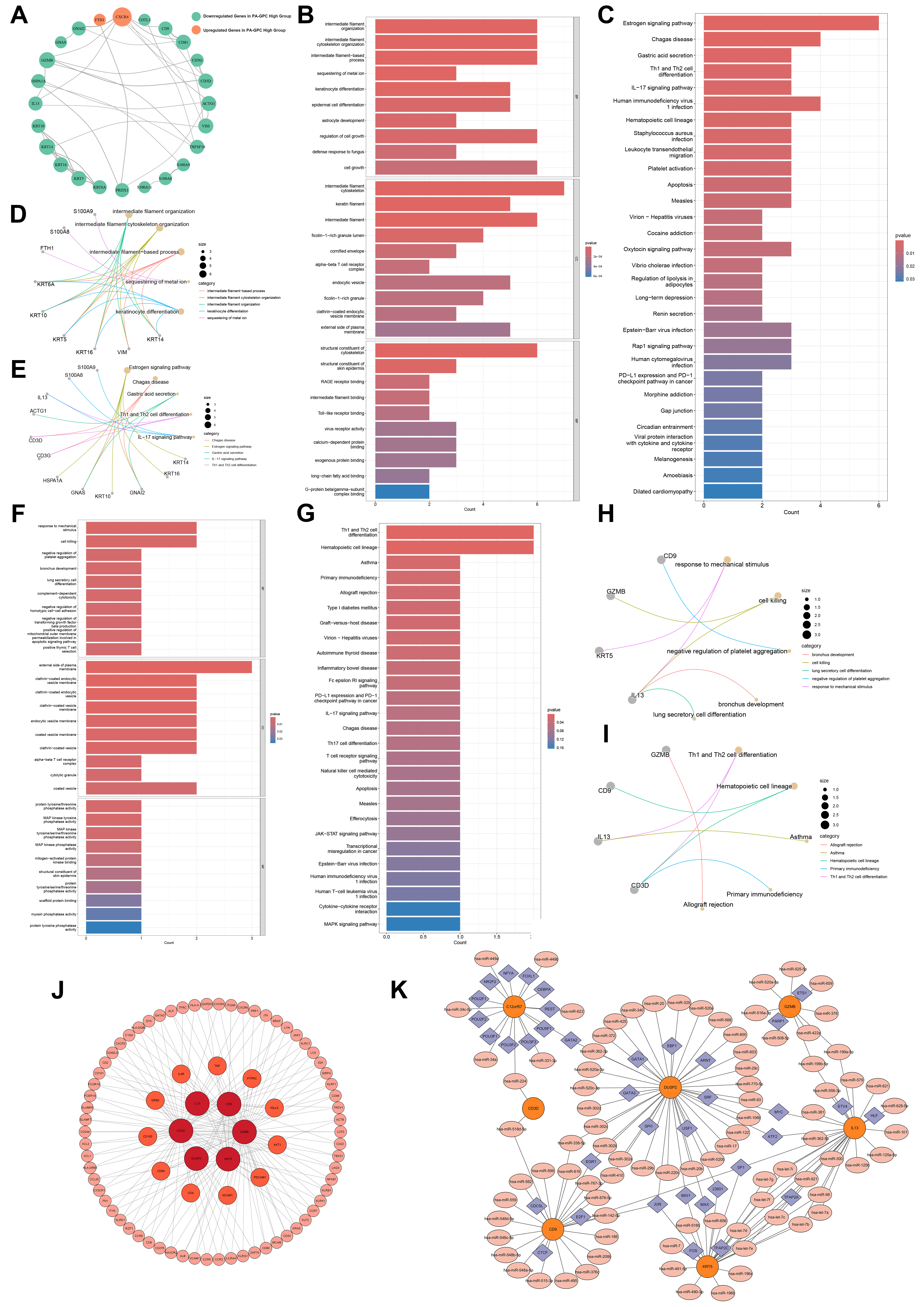


**Figure S2. Functional interaction networks and enrichment analysis of PA-GPC–associated differentially expressed and hub genes.**

**(A) Protein–protein interaction (PPI) network constructed from the 33 PA-GPC–associated differentially expressed genes (DEGs). (B)** GO enrichment analysis of the 33 DEGs. **(C)** KEGG pathway enrichment analysis of the 33 DEGs. **(D-E) Cnetplots illustrating gene–function relationships based on Gene Ontology (GO) (D) and KEGG pathway (E) enrichment analyses of the 33 DEGs.** **(F–G)** GO (F) and KEGG (G) enrichment analyses of the hub genes.
**(H–I)** Cnetplots showing gene–function relationships based on GO (G) and KEGG (H) results. **(J)** PPI network of hub genes. **(K)** Transcription factor and microRNA regulatory network of hub genes.


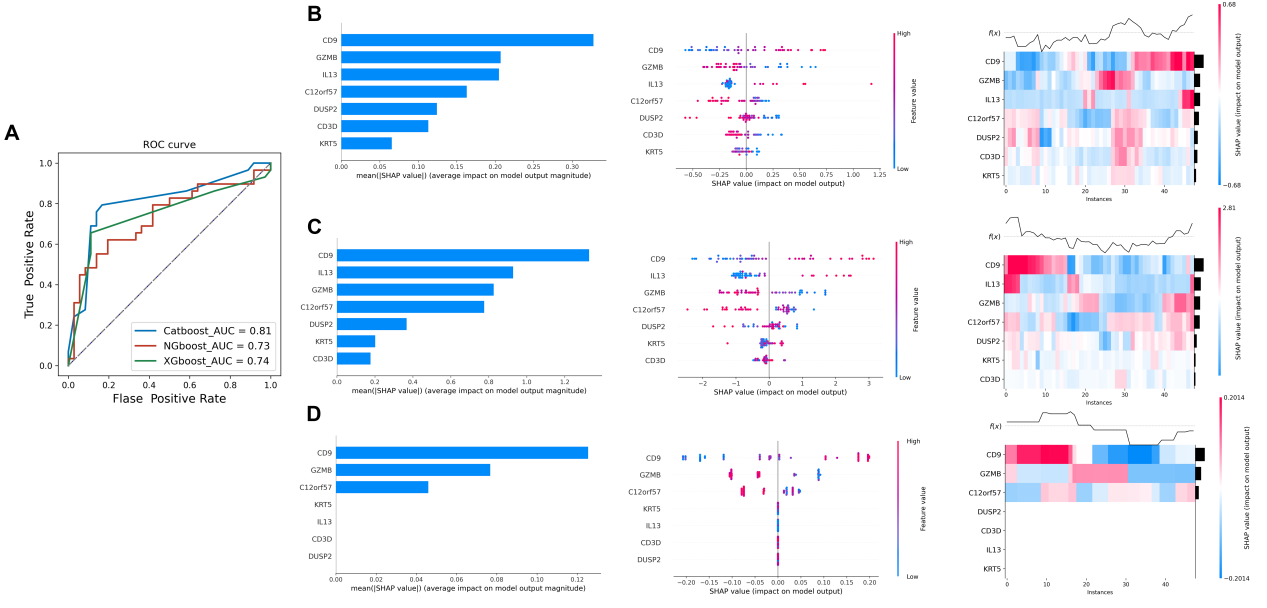


**Figure S3. Machine learning model validation and SHAP analyses in the second cohort.**

**(A)** ROC curves for three machine learning models (CatBoost, NGBoost, XGBoost) based on hub gene expression in predicting AD. **(B–D)** SHAP analysis showing the importance of individual genes in the CatBoost (B) , NGBoost (C) , XGboost(D) models, including bar plots, beeswarm plots, and heatmaps.


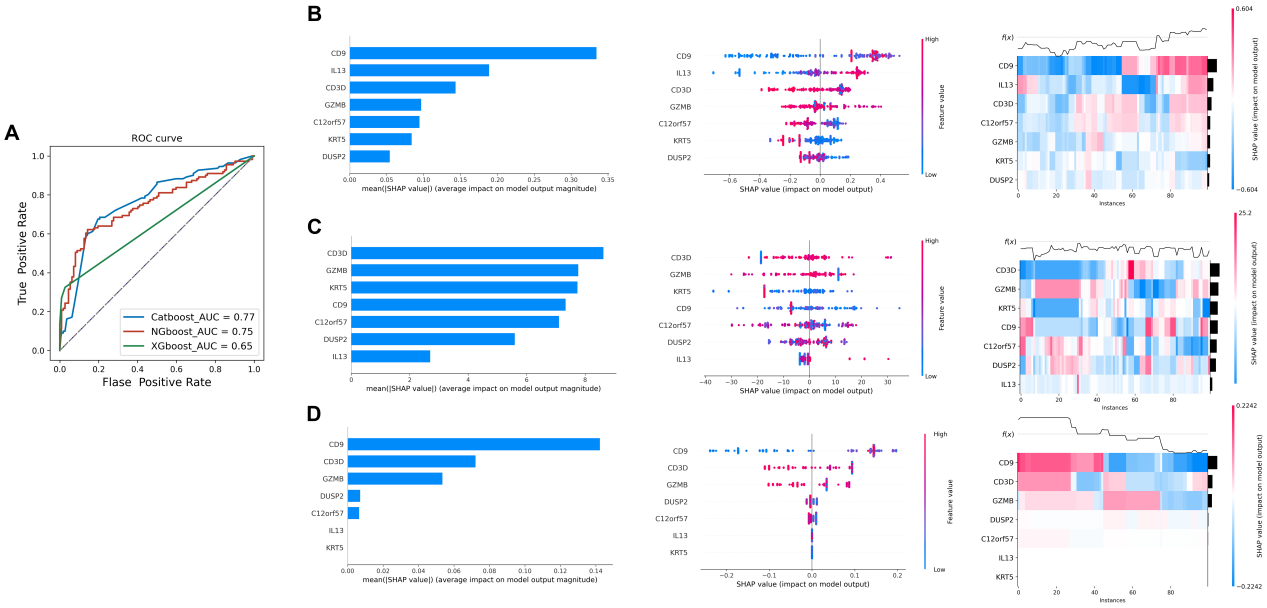


**Figure S4. Machine learning model validation and SHAP analyses in the third cohort.**

**(A)** ROC curves for three machine learning models (CatBoost, NGBoost, XGBoost) based on hub gene expression in predicting AD. **(B–D)** SHAP analysis showing the importance of individual genes in the CatBoost (B) , NGBoost (C) , XGboost(D) models, including bar plots, beeswarm plots, and heatmaps.
